# Proteomics analysis in myocardium of spontaneously hypertensive rats

**DOI:** 10.1038/s41598-023-27590-8

**Published:** 2023-01-06

**Authors:** Tingjun Wang, Xiaoqi Cai, Jinze Li, Liangdi Xie

**Affiliations:** 1 Department of General Practice, The First Affiliated Hospital, Fujian Medical University, Fuzhou, 350005 People’s Republic of China; 2 The International Medical Services, National Regional Medical Center, Binhai Campus of the First Affiliated Hospital, Fujian Medical University, Fuzhou, 350005 People’s Republic of China; 3 Department of Geriatrics, The First Affiliated Hospital, Fujian Medical University, Fuzhou, 350005 People’s Republic of China; 4 Fujian Hypertension Research Institute, Fuzhou, 350005 People’s Republic of China; 5 Branch of National Clinical Research Center for Aging and Medicine, Fujian Provincial Clinical Research Center for Geriatric Hypertension Disease, National Clinical Research Center for Aging and Medicine, Huashan Hospital, Fudan University, Fuzhou, 350005 Fujian People’s Republic of China

**Keywords:** Cardiology, Molecular medicine

## Abstract

Hypertension-related left ventricular hypertrophy is recognized as a good predictor of adverse cardiovascular events. However, the underlying mechanism of left ventricular hypertrophy is still not fully understood. This study employed liquid chromatography coupled with tandem mass spectrometry to investigate global changes in protein profile in myocardium of spontaneously hypertensive rat, a classical animal model of essential hypertension. There were 369 differentially expressed proteins in myocardium between spontaneously hypertensive rats and normotensive rats. Xenobiotic catabolic process, cholesterol binding and mitochondrial proton-transporting ATP synthase were found to be the most significantly enriched biological process, molecular function and cellular component terms of Gene Ontology, respectively. Drug metabolism-cytochrome P450 was revealed to be the most significantly enriched Kyoto Encyclopedia of Genes and Genomes pathways. FYN proto-oncogene, Src family tyrosine kinase was found to have the most interactions with other proteins. Differentially expressed proteins involved in xenobiotic catabolic process, lipid transport and metabolism, mitochondrial function might be targets for further study of hypertension-related left ventricular hypertrophy.

## Introduction

Hypertension is the most common chronic disease worldwide, and left ventricular hypertrophy (LVH) is recognized as a primary target for hypertension end-organ damage, and it has been proved to be a good predictor of adverse cardiovascular events^1^. As a complicated pathological process, LVH may result from the changes of numerous proteins. Proteomics analysis is a potential approach to identify, characterize and quantify global changes in protein profile, and the approach has been applied to examine protein profiles in myocardium of spontaneously hypertensive rats (SHRs), an inbred homozygous rodent model of human essential hypertension^2^. However, the sample separation technologies used in previous studies are almost all two-dimensional polyacrylamide gel electrophoresis (2D PAGE). Although 2D PAGE is widely used in proteomics research in the past, it is far from a perfect approach. There are many disadvantages in this technology. Firstly, it is difficult to detect low abundant proteins, only proteins with high abundance can be detected by 2D PAGE. Secondly, hydrophobic proteins are hardly separated on 2D PAGE. Thirdly, extremely acidic or basic proteins or proteins with molecular weight less than 10 kDa can not be observed on 2D PAGE. In practice, membrane proteins and small proteins are often missed out, and 2D PAGE can visualize only 30–50% of entire proteome^3^. In addition, 2D PAGE is labor-intensive and time-consuming^3^. Obviously, it is considered unsuitable for high throughput and large-scale proteomics analysis. At present, liquid chromatography (LC) has emerged as a powerful technique which allows continuous separation of thousands of proteins, and can be performed online with mass spectrometry (MS) for higher throughput^4^. In this study, LC coupled with tandem MS (LC–MS/MS) was used to detect the changes of protein expression in myocardium of adult SHRs.

## Results

### Blood pressure, left ventricle relative mass, hemodynamic parameters of left ventricle

Systolic blood pressure was significantly higher in SHRs than in Wistar Kyoto rats (WKYs ) (192.0 ± 7.4 vs 130.8 ± 7.0 mmHg, *P* < 0.001). Left ventricle relative mass was markedly increased in SHRs compared with WKYs (0.41 ± 0.03 vs. 0.32 ± 0.19 g/100 g, *P* < 0.001). There was no difference in heart rate and maximum rate of pressure decline (− dp/dt max) between two strains of rats. However, SHRs displayed elevated left ventricular end-systolic pressure (LVESP) and end-diastolic pressure (LVEDP), maximum rate of pressure rise (+ dp/dt max) (Table 1).

**Table 1:** Comparison of heart rate and hemodynamic parameters of left ventricle between SHRs and WKYs(*n* = 5).

	SHR	WKY	*t*	*P*
Heart rate (beats/min)	406 ± 16	390 ± 22	1.362	0.210
LVESP (mmHg)	202.2 ± 14.8	132.8 ± 9.5	9.062	< 0.001
LVEDP (mmHg)	9.1 ± 0.2	4.1 ± 0.2	40.335	< 0.001
LV + dp/dt _max_ (mmHg/s)	6940 ± 472	5128 ± 463	6.130	< 0.001
LV − dp/dt _max_ (mmHg/s)	4166 ± 128	4070 ± 120	1.224	0.256

### Quality control

The mass deviations of all identified peptides were mainly distributed within 10 ppm, indicating the identification was accurate and reliable (supplementary Figure S1a online). MASCOT score was relatively high, about 70.17% of peptides scored above 20, the median of peptide score was 31.01 (supplementary Figure S1b online). For tandem mass tag (TMT) data, false discovery rate (FDR) ≤ 0.01 was set as the screening criterion of qualitative analysis. These data implied that MS quality was good. The distribution ratio of most proteins between two sets of samples was close to 1 (supplementary Figure S1c).

### Identification of differentially expressed proteins (DEPs) in left ventricle between SHRs and WKYs

**Figure 1:**
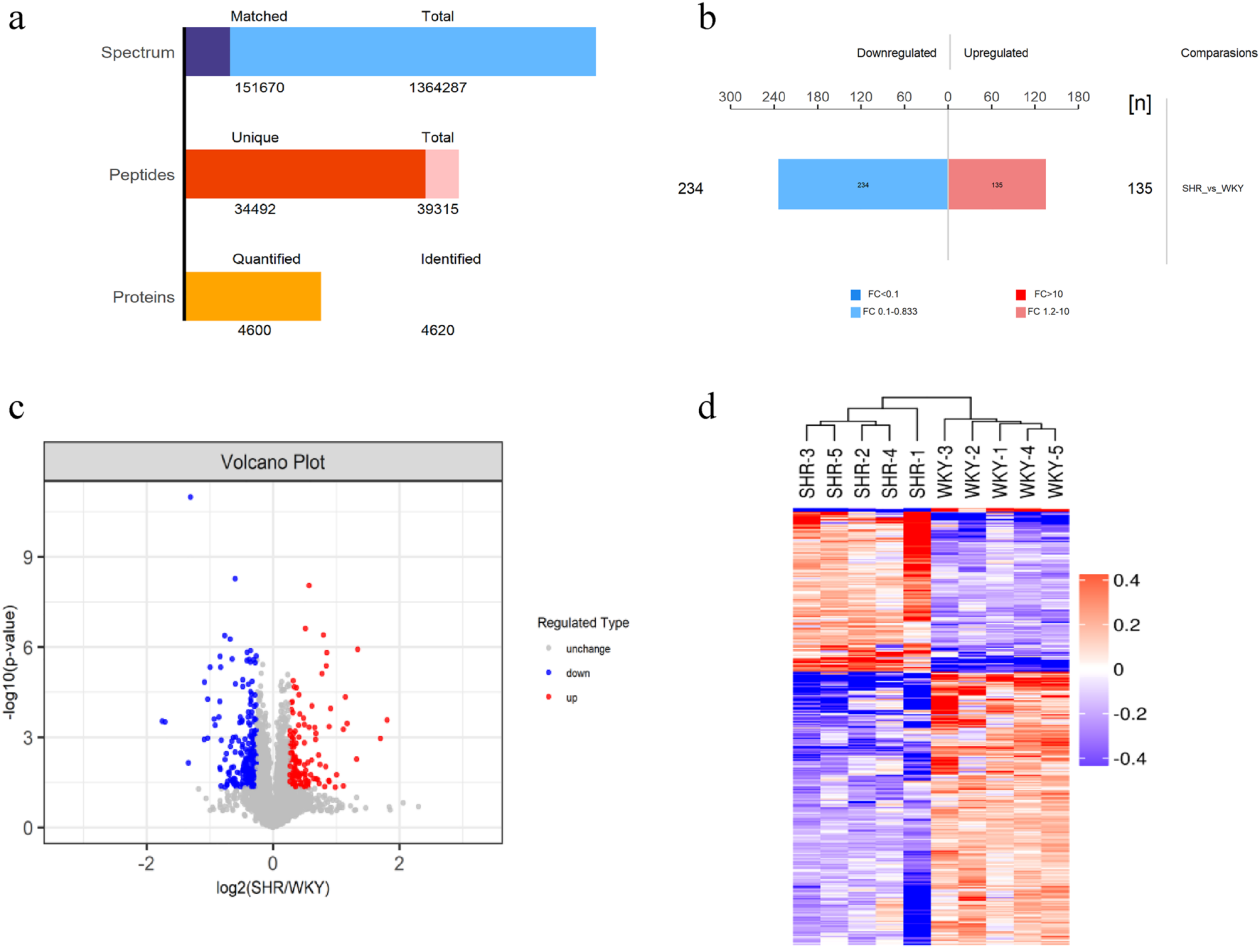
Identification and quantification of peptides and proteins in left ventricle of SHRs and WKYs. (**a**) peptides and proteins. (**b**) The analysis of DEPs. (**c**) Volcano plots of quantified proteins. (**d**) Hierarchical clustering analysis of DEPs.

A total of 4620 proteins were identified in the present study, in which 4600 proteins were quantified (Fig. 1a). Differential expression analysis revealed that 369 proteins differed significantly in abundance between two strains of rats, including 135 upregulated proteins and 234 downregulated proteins in myocardium of SHRs (Fig. 1b,c). Further hierarchical clustering analysis showed that SHRs were distinguished from WKYs by these DEPs (Fig. 1d).

The top 10 proteins with the most significant difference in abundance are presented in Table 2, of which 6 proteins (LRGUK, VHL, ADGRA3, MAN2C1, ZFP494, SIRPA) were upregulated and 4 proteins (ENDOG, HBA-A3, SERHL2, FMO2) were downregulated.

**Table 2:** The top 10 differentially expressed proteins in left ventricle between SHRs and WKYs.

Protein	Description	Number of unique peptides	Sequence coverage (%)	SHR/WKY	*P*
LRGUK	Leucine-rich repeats and guanylate kinase domain containing	1	0.85	3.486	< 0.001
VHL	Von Hippel-Lindau tumor suppressor	1	7.03	2.494	0.005
ADGRA3	Adhesion G protein-coupled receptor A3	1	0.54	2.254	< 0.001
MAN2C1	Mannosidase, alpha, class 2C, member 1	1	1.45	2.161	< 0.001
ZFP494	Zinc finger protein 494	1	8.13	2.160	0.042
SIRPA	Signal-regulatory protein alpha	2	3.73	2.007	0.018
ENDOG	Endonuclease G	10	41.16	0.404	< 0.001
HBA-A3	hemoglobin alpha, adult chain 3	4	42.96	0.471	0.001
SERHL2	serine hydrolase-like 2	4	13.55	0.472	< 0.001
FMO2	flavin containing monooxygenase 2	3	5.61	0.502	< 0.001

**Figure 2:**
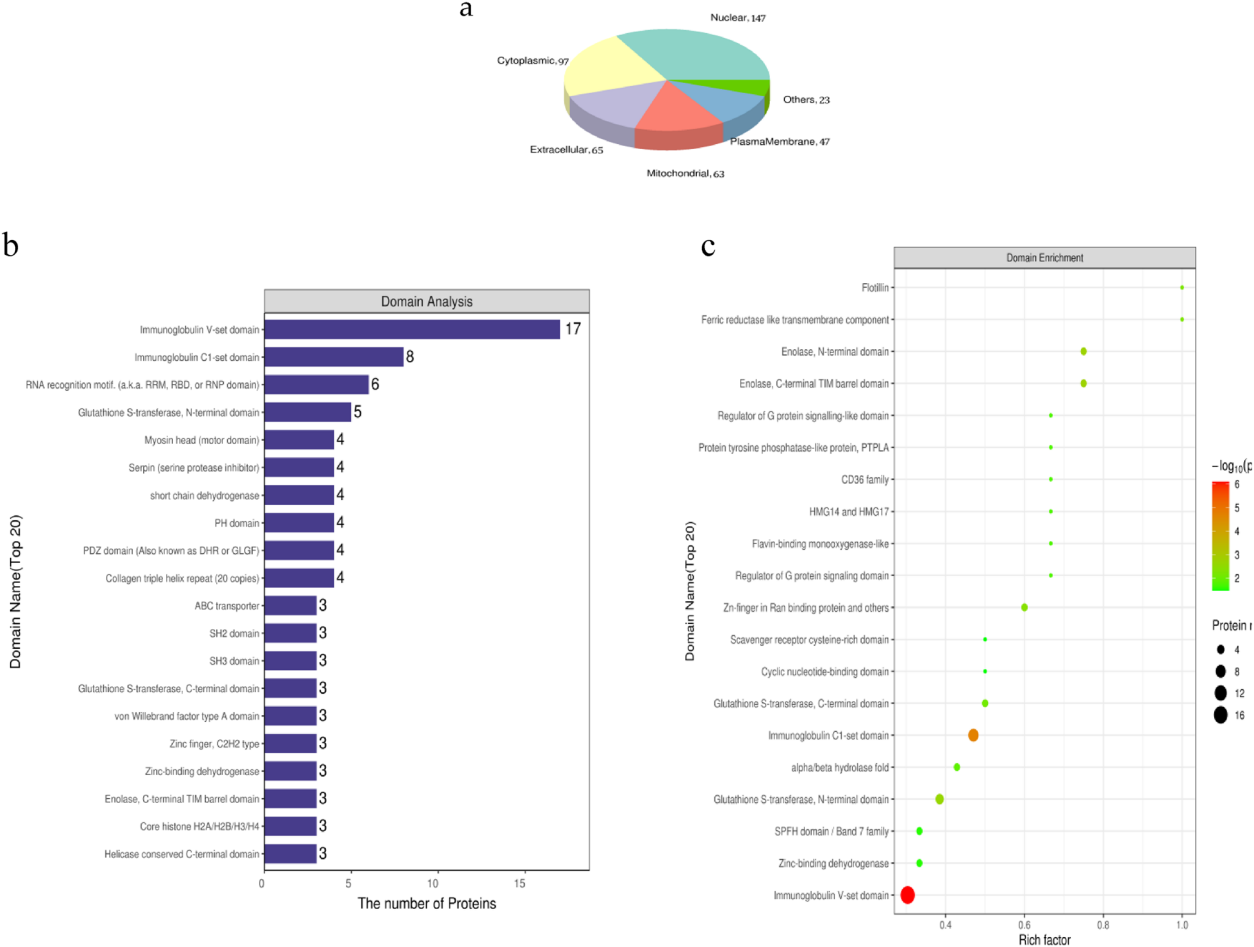
Subcellular location, domain characteristics and GO enrichment analysis of the DEP domains in left ventricle between SHRs and WKYs. (**a**) Subcellular location. (**b**) The top 20 domains. (**c**) Bubble chart of the top 20 enriched domains. The ordinate represented domain names. The abscissa represented rich factor, rich factor was defined as the number of DEPs annotated in the domain name divided by the total number of all identified proteins in the corresponding domain name. The color of bubble indicated the significance of enriched domain name and the color closer to red indicated greater significance.

It was found that the DEPs were predominantly located in nuclear, followed by cytoplasmic, extracellular and mitochondrial domains and then plasma membrane (Fig. 2a). Of note, there were 63 DEPs located in mitochondrial domain, the number was very large for the mitochondrial with the total number of proteins less than 2000. Immunoglobulin V-set was shown to be the most frequently observed domain in amino acid sequences of all DEPs (Fig. 2b). Similarly, this sequence was the most significantly enriched domain in enrichment analysis of DEPs (Fig. 2c).

### Gene ontology (GO) annotation and enrichment analysis of DEPs

**Figure 3:**
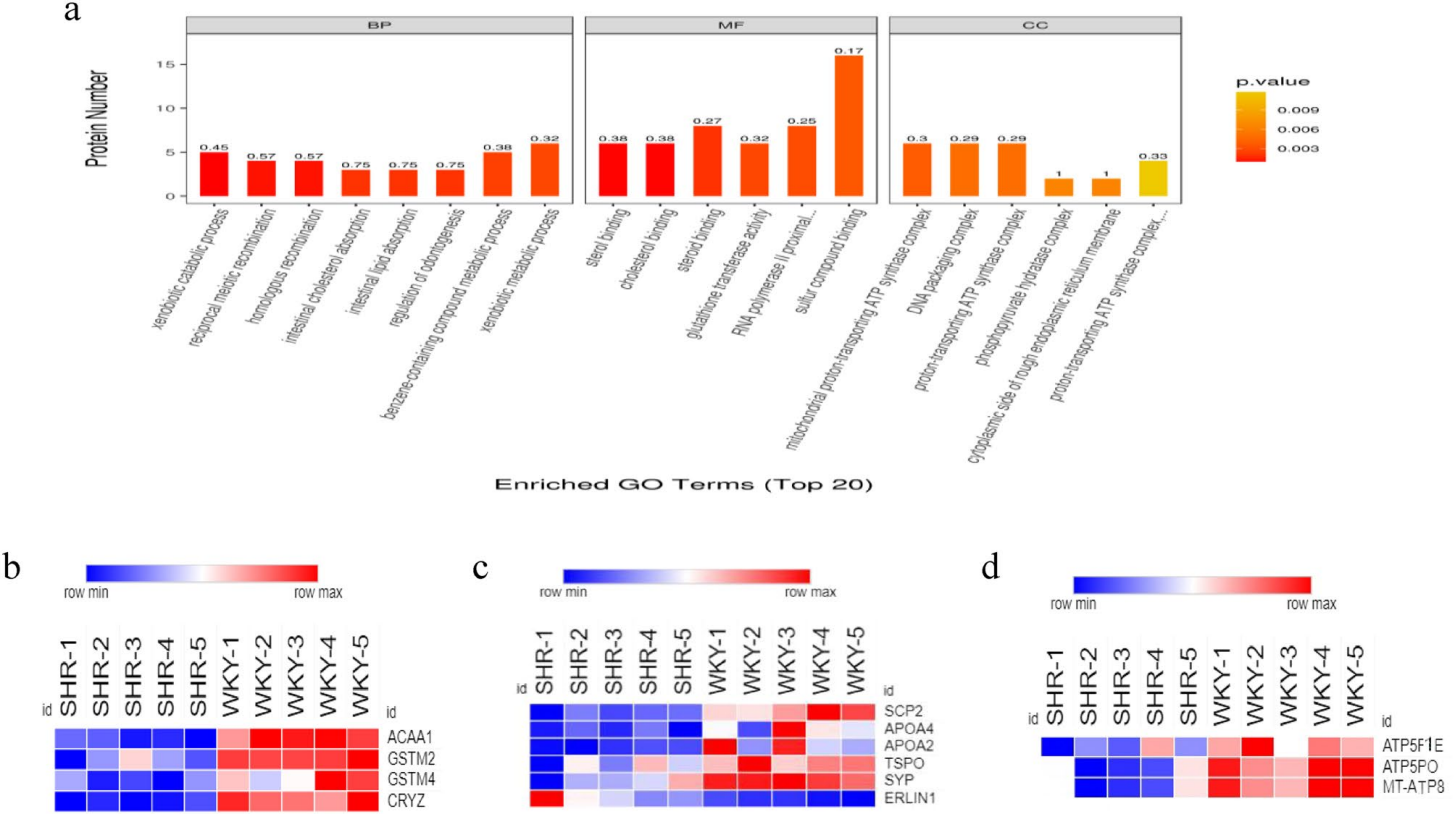
GO enrichment analysis of DEPs according to biological process (BP), molecular function (MF), cellular component (CC) terms. (**a**) GO enrichment of DEPs. (**b**, **c**, **d**) Hierarchical clustering analysis of the expression of DEPs annotated in xenobiotic catabolic process, cholesterol binding, mitochondrial proton-transporting ATP synthase, proton-transporting ATP synthase, DNA packaging complex terms.

Xenobiotic catabolic process was shown to be the most prominently enriched GO biological process (Fig. 3a), and DEPs in xenobiotic catabolic process included ACAA1A, GSTM2, GSTM4, CRYZ (Fig. 3b). Cholesterol binding was the most significantly enriched GO molecular function (Fig. 3a), SCP2, ERLIN1, TSPO, SYP, APOA2 and APOA4 were the DEPs annotated in cholesterol binding (Fig. 3c). Mitochondrial proton-transporting ATP synthase, proton-transporting ATP synthase, DNA packaging complex were the most markedly enriched GO cellular component terms (Fig. 3a), ATP5F1E, ATP5PO and MT-ATP8 were overlapped DEPs annotated in the three cellular component terms (Fig. 3d).

### Kyoto encyclopedia of genes and genomes (KEGG) enrichment analysis and PPI analysis of DEPs

**Figure 4:**
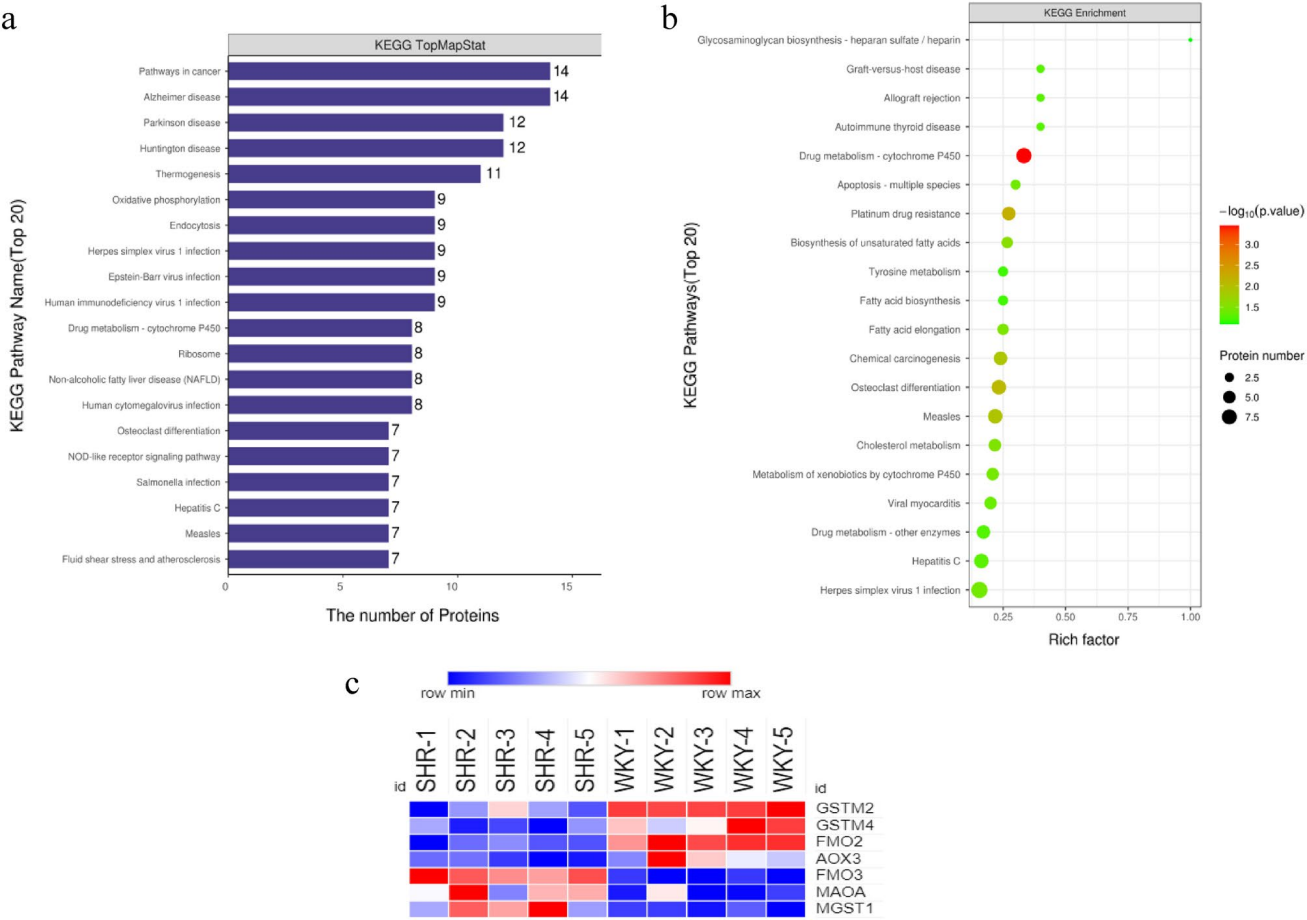
KEGG pathway annotation^6^ and enrichment analysis of DEPs in left ventricle between SHRs and WKYs. (**a**) The top 20 terms of KEGG pathways with the largest number of DEPs. (**b**) Bubble chart of the top 20 enriched KEGG pathways. (**c**) The expression of DEPs in drug metabolism-cytochrome P450.

**Figure 5:**
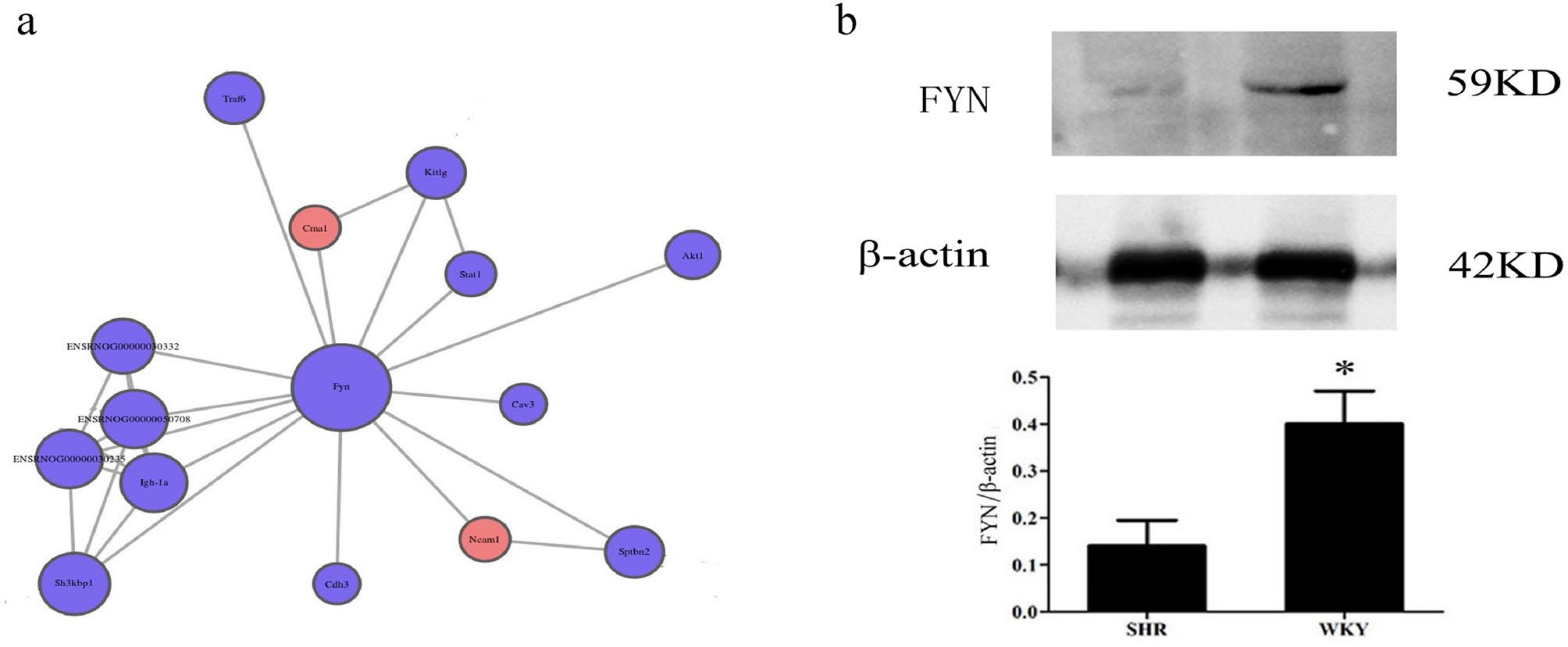
Protein–protein interaction of FYN. (**a**) Protein–protein interaction, blue circles indicated downregulated DEPs, and red circles indicated upregulated DEPs; (**b**) Representative western blot image and the ratio of FYN to β-actin. Original blots are included in a supplementary Figure S2 online. ^*^P < 0.05.

Pathways in cancer, Alzheimer’s disease, Parkinson disease were the KEGG pathways with the largest number of DEPs (Fig. 4a). Drug metabolism-cytochrome P450 was the most significantly enriched KEGG pathways (Fig. 4b). The DEPs annotated in this pathway included GSTM2, GSTM4, MGST1, FMO2, AOX3, FMO3, MAOA (Fig. 4c). In protein–protein interaction (PPI) analysis, it was shown that FYN was the hub protein with 14 interactions with other proteins (Fig. 5a). The downregulated expression of FYN in myocardium of SHRs was verified by western blot (Fig. 5b).

## Discussion

In this study, LC–MS/MS was used to investigate the proteome changes in left ventricle in the animal model of essential hypertension. It was found that there were 369 DEPs in left ventricle between normotensive and hypertensive rats. Further analysis showed xenobiotic catabolic process, cholesterol binding and mitochondrial proton-transporting ATP synthase were the most prominently enriched GO biological process, molecular function and cellular component terms, respectively. Drug metabolism-cytochrome P450 was the most significantly enriched KEGG pathways. The data indicated that some proteins involved in xenobiotic catabolic process, lipid transport and metabolism, mitochondrial function might play a vital role in hypertension-related LVH.

Adult SHR is an ideal animal model of compensatory LVH, as supported by increased left ventricle relative mass, elevated left ventricular end-systolic pressure in combined with greater + dp/dt max in SHR. As expected, more DEPs were found in this study than previous study possible due to the adoption of LC–MS/MS^2^. It is noteworthy that a great number of DEPs were located in mitochondria, implying the vital role of mitochondrial dysfunction in hypertension-related LVH. In the top 10 DEPs, ENDOG has been reported to play an important role in LVH development^5^. ENDOG is a nuclear-encoded, mitochondria-localized nuclease^6^. Decreased ENDOG is observed in all stains of rats that have LVH^6^. Mitochondrial dysfunction and elevated levels of reactive oxygen species (ROS) are proved to be the mechanisms underlying LVH caused by deletion of *Endog*^7^.

Notably, xenobiotic catabolic process was the top enriched GO biological process term in the present study. Generally, the DEPs annotated in xenobiotic catabolic process have the capacity of oxidation or reduction, and the enrichment of this term often implies the imbalance in redox system^8^. As DEPs in this process, ACAA1A catalyses the final step in beta-oxidation of fatty acid and degradation of branched-chain amino acid through mediating the reversible conversion of acetoacetyl-CoA to acetyl-CoA^8^. ACAA1A is also reported to mediate the first step in the mevalonate pathway, and the end-product of this pathway is a precursor for cholesterol^9^. Therefore, ACAA1A is regarded as a key enzyme at the crossroad of metabolic pathways that respond to the cellular energy status. Cardiac hypertrophy is characterized by decreased fatty acid beta-oxidation and increased glycolysis, which seem consistent with the downregulated expression of ACAA1A in myocardium of SHR^10^. Cholesterol binding was found to be the most significantly enriched GO molecular function. In the DEPs annotated in cholesterol binding, SCP2 has long been known as the non-specific lipid transfer proteins^11^. Although it is annotated to cholesterol binding in GO biological process term, it is currently considered to function as a carrier of fatty acyl-CoAs in peroxisomal fatty-CoA metabolism rather than cytosolic sterol transport, as evidenced by a defect in peroxisomal β-oxidation and diminished peroxisomal α-oxidation of phytanic acid in SCP null mice^12,13^. ERLIN1 is a cholesterol-binding protein located in endoplasmic reticulum, and it interacts with ERLIN2 to form a ring-shaped complex which is involved in endoplasmic reticulum-associated degradation^14^. ERLIN1 is also proved to participate in the regulation of cholesterol homeostasis and fatty acid biosynthesis through stabilizing SREBP-Scap-Insig complex^15^. TPSO is also identified as cholesterol binding protein, and it is primarily localized in outer mitochondrial membrane^16^. In addition to regulating steroidogenesis, TSPO is found to be involved in a wide variety of mitochondrial physiology and metabolism including modulation of anion channels, adenosine triphosphate (ATP) production, ROS generation and release, regulation of mitochondrial membrane potential and mitochondrial respiratory chain^17^. It is reported that TPSO knockout protects against pressure overload induced cardiac hypertrophy by preserving mitochondrial function^18^. The most markedly enriched GO cellular component term is mitochondrial proton-transporting ATP synthase, which is also known as mitochondrial complex V. DEPs in this term belong to the subunits of the complex, and the changes in the expression of subunits indicate diminished ATP production in myocardium of SHR. DEPs in this term belong to the subunits of the complex, and the changes in the expression of subunits indicate diminished ATP production in myocardium of SHR. Taken together, the dysregulation of redox system, lipid transport and metabolism as well as mitochondria function represent the major characteristics of pathological process of hypertension-related LVH, the DEPs annotated in these terms might be targets for further study.

In the analysis of KEGG pathways, enrichment analysis showed that drug metabolism-cytochrome P450 was the most significantly enriched KEGG pathway. Generally, most of DEPs in this pathway are also related to the balance between oxidant stress and anti-oxidant capacity^19,20^. Consequently, it is proved again that cardiac hypertrophy is closely linked to redox imbalance. Additonally, Alzheimer’s disease was observed to be the pathways with the larger number of DEPs. Nowadays, cardiac hypertrophy caused by hypertension is regarded as “Alzheimer’s”of the heart^21^. Our data support this claim.

FYN is found to be one of hub proteins. As a non-receptor tyrosine-protein kinase, FYN plays a role in various biological processes including regulation of cell growth and survival, cell adhesion, integrin-mediated signaling, cytoskeletal remodeling, cell motility^22^. In myocardium, FYN is reported to regulate sodium channel and inhibit ROS production^23,24^. FYN expression was verified to be downregulated in myocardium of SHRs. Therefore, its role needs to be investigated.

A major strength was that more DEPs were identified using LC–MS/MS, which help us comprehensively understand molecular changes in hypertension-related LVH. However, some limitations must be acknowledged. The proteome of left ventricle was analyzed at only one time point in the duration of life in rats. Therefore, it is impossible to fully understand the proteome changes during LVH progression, primary and compensatory changes in protein expression can not be determined. Additionally, an intact myocardium rather than specific component in left ventricle was chosen for proteomics analysis, a specific location will be required for interested DEPs in future research.

In this study based on SHR, it is deduced that the changes of proteins involved in xenobiotic catabolic process, lipid transport and metabolism, mitochondrial function may be key characteristics of proteomics in the hypertension-related LVH. Proteomics analysis using LC–MS/MS provides a greater insight into the mechanism underlying hypertension-related LVH.

## Methods

### Animals, blood Pressure and hemodynamic measurements and tissue collection

Five male SHRs and five male WKYs at the age of 20 weeks (Vital River Laboratory Animal Technology Co., Ltd., Beijing, China, Certificate No.2012–0001) were used in this experiment. Bodyweight was determined using an electronic scale, and systolic blood pressure was measured using a tail-cuff method (Softron BP-98a; Softron Co., Ltd., Tokyo, Japan), as previously described^25^. Then, rats were anaesthetized with sodium pentobarbital anaesthesia (50 mg/kg, administered intraperitoneally), and a pressure transducer (Science FT211B of 1.6F diameter) was introduced from right carotid artery to left ventricle. The catheter was connected to a data acquisition system (PowerLab/800, AD Instruments). Signals were monitored and automatically stored for further analysis. After hemodynamic measurement, the animals were sacrificed by decapitation and left ventricle including ventricular septum was removed and weighed. Left ventricle relative mass was calculated by left ventricle mass divided by body weight (g/100 g). The myocardium at the apex (20 g) was stored at − 80 °C for proteomics analysis and western blot. The study protocol was approved by the Laboratory Animal Welfare and Ethics Committee of Fujian Medical University (approval No. 2014001), and all procedures were carried out in accordance with the ethical standards, all methods are reported in accordance with ARRIVE guidelines.

### Extraction and digestion of protein, labeling and fraction of peptide, LC–MS/MS analysis and data Processing

Protein was extracted using SDT (4%SDS, 100 mM Tris–HCl, 1 mM DTT, pH 7.6) buffer. The concentration was determined by BCA Protein Assay Kit (Bio-Rad, California, USA), and extracted protein was digested by trypsin and desalted on C18 Cartridges. The peptides were further centrifuged and reconstituted in 40 µl of 0.1% (v/v) formic acid. Peptide was labeled (100 μg/sample) with TMT reagent and further fractionated by High pH Reversed-Phase Peptide Fractionation Kit (Thermo Scientific, Massachusetts, USA). Subsequently, the fractionated peptide mixture was reconstituted and acidified with 0.1% TFA solution, following by being loaded to the equilibrated, high-pH, reversed-phase fractionation spin column. Peptides were bound to resin and desalted by washing the column. Bound peptides were eluted by step gradient, step gradient was established by increasing concentrations of acetonitrile in a volatile high-pH elution solution. Ten different fractions were collected by a series of steps including centrifugation and desalination and vacuum centrifugation. The concentrated fractions of the labeled peptides were loaded onto a C18 trap column in 0.1% formic acid and separated with a linear gradient. The mass spectrometer (A Q Exactive mass spectrometer coupled with Easy nLC for 60/90 min, Thermo Scientific, Massachusetts, USA) was operated in positive ion mode. MS1 and MS2 spectra were obtained using data-dependent top 10 method (300–1800 m/z). MASCOT (Matrix Science, London, UK; version 2.2) and Proteome Discoverer 1.4 software were used to search and analyze the data of MS spectrum.

### Bioinformatics analysis

DEPs were defined as the proteins with fold changes in the expression between SHRs and WKYs > 1.2 and *P* < 0.05. Hierarchical clustering analysis of DEPs was performed using Cluster 3.0, Java Treeview software or Morpheus. Volcano plot and heat maps were presented as a visual aid. NCBI BLAST + client software and InterProScan were used to search homologue sequences of DEPs. Subcellular location and domain characteristics of DEPs were analyzed using CELLO and InterProScan. GO enrichment analysis and KEGG pathways enrichment analysis of DEPs were performed using Blast2GO, InterProScan and KAAS (KEGG Automatic Annotation Server), respectively. PPI of DEPs was acquired from IntAct or STRING database, and the information was further analyzed by CytoScape software (version 3.2.1).

### Western-blot analysis

Aliquots of 80 μg protein were separated by 10% SDS-PAGE, electro-transferred onto polyvinylidene fluride membranes (EMD Millipore), blocked with 5% non-fat milk in TBS containing 0.05% Tween-20 (TBST). The membranes were cut prior to hybridization and incubated overnight with the following antibody, respectively: anti-FYN antibody (1:200, ab184276, abcam, Cambridge, UK) and anti-β-actin antibody (1:1000, sc1616, Santa Cruz Biotechnology Inc.Dallas, TX, USA). After three washes with TBST, the blot was hybridized with secondary antibody at room temperature for 1 h. Protein bands were detected by ECL western blotting substrate and quantified using Image Pro Plus version 6.0 (Media Cybernetics,Inc.Maryland, USA).

### Statistical analysis

Blood pressure, hemodynamic data and the data of western blot are expressed as mean ± standard deviation (SD). The comparison was performed using *t*-test (SPSS 20.0 software package, SPSS, Inc., Chicago, IL, USA). Enrichment analysis was determined based on Fisher’s exact *t* test, and *P* < 0.05 is considered significant.

## Supplementary Information

Supplementary Information.

## Data Availability

The sources of all data used in this study are referenced in the “Methods” section and all datasets used for the analyses are available from the corresponding author upon reasonable request.
