# Supplementary material for: Proteomics analysis in myocardium of spontaneously hypertensive rats
Source: Sci Rep. 2023 Jan 6;13:276. doi: 10.1038/s41598-023-27590-8 (PMC9822958; doi:10.1038/s41598-023-27590-8)
Supplement: Supplementary file 1 — Supplementary Information. [file 41598_2023_27590_MOESM1_ESM.pdf]

Proteomics analysis in myocardium of spontaneously hypertensive rats

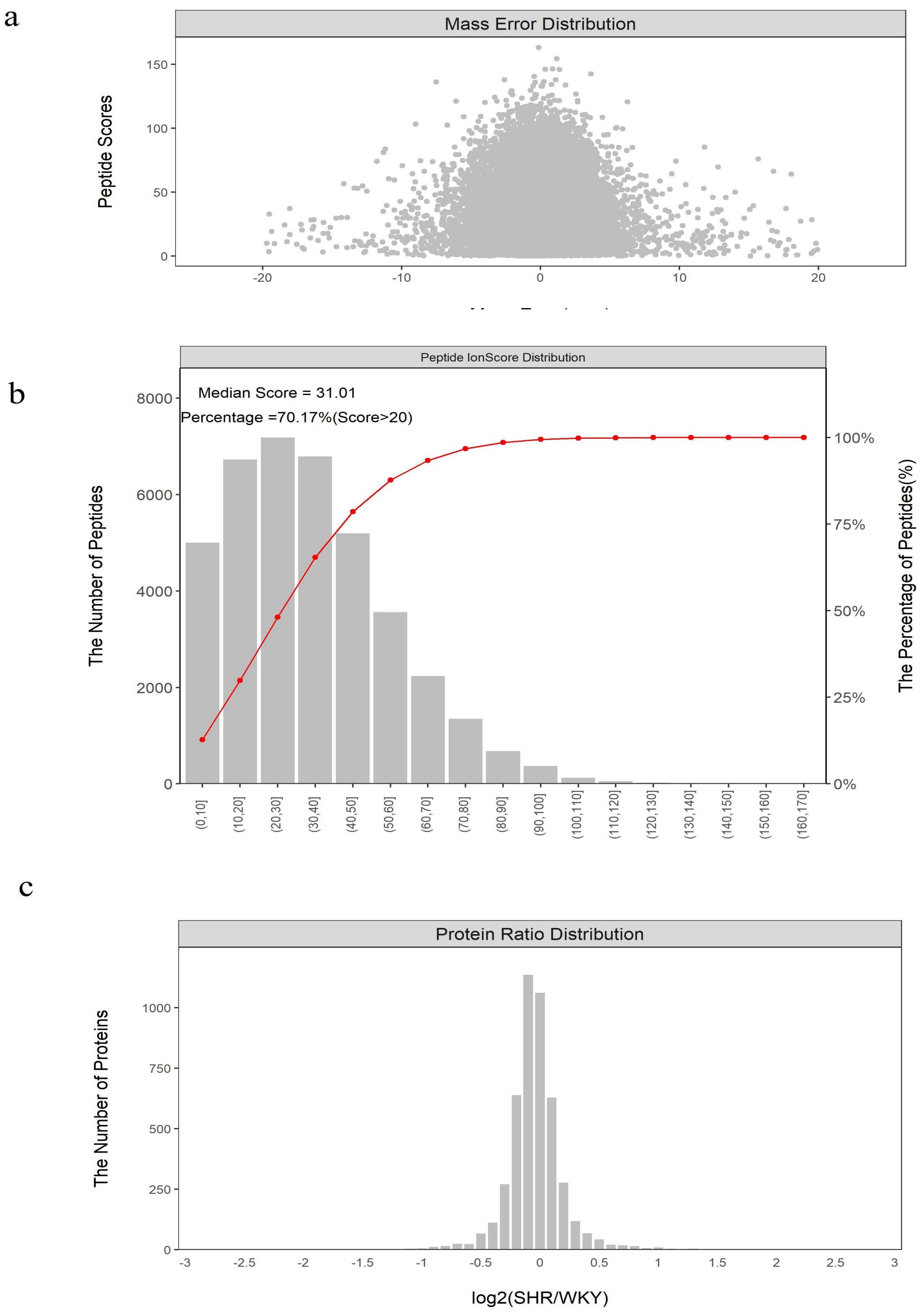

Figure S1 The data of quality control. (**a**)The mass deviations of all identified peptides. (**b**) A score for each MS2 spectrum. (**c**) Protein ratio distribution.

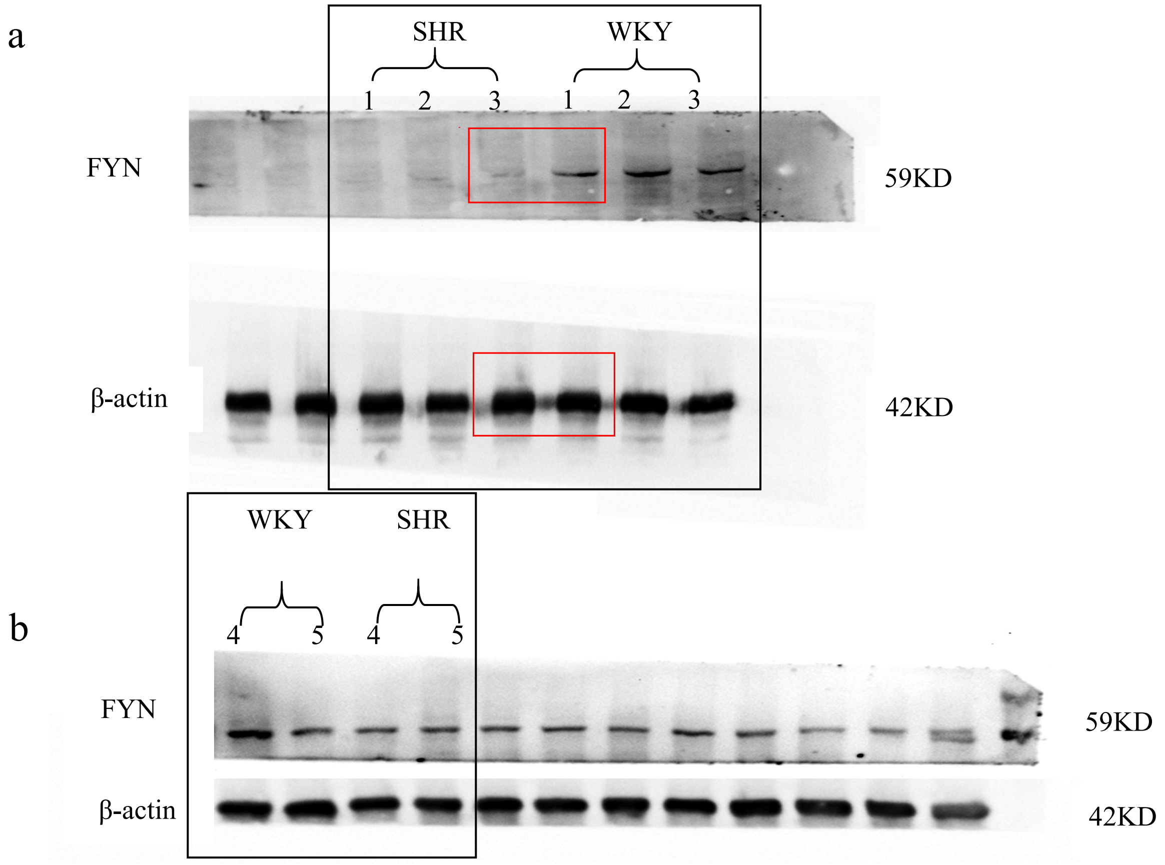

Figure S2 Original western blot image. The polyvinylidene fluride membranes were cut prior to hybridization with antibodies during blotting. The number represents the rat identifier, and representative western blot images were in red box. The blots of experimental samples in this study were in black box.
